# Point-of-care ultrasound in dogs with clinical signs due to heartworm disease

**DOI:** 10.1186/s13071-026-07720-7

**Published:** 2026-10-21

**Authors:** Marisa K. Ames

**Affiliations:** Department of Medicine and Epidemiology, Veterinary Medical Teaching Hospital, University of California, Davis, CA USA

**Keywords:** Pneumonitis, Pulmonary hypertension, Congestive heart failure, Caval syndrome

## Abstract

Estimates suggest that over 1 million dogs in the United States are infected with heartworm (*Dirofilaria immitis*). Although dogs often have no, or only mild clinical signs with heartworm infection, some dogs develop life-threatening cardiopulmonary disease. Point-of-care ultrasound is a useful tool to assess dogs with severe heartworm disease and varies from a quick ‘fluid check’ to more comprehensive exams that utilize standardized views. Ultrasound is commonly used to diagnose cavitary effusions and intracardiac heartworms. With more advanced training, ultrasound can be used to identify interstitial lung abnormalities and evaluate the morphology of the right heart.

**Graphical Abstract:**

**Figure:**
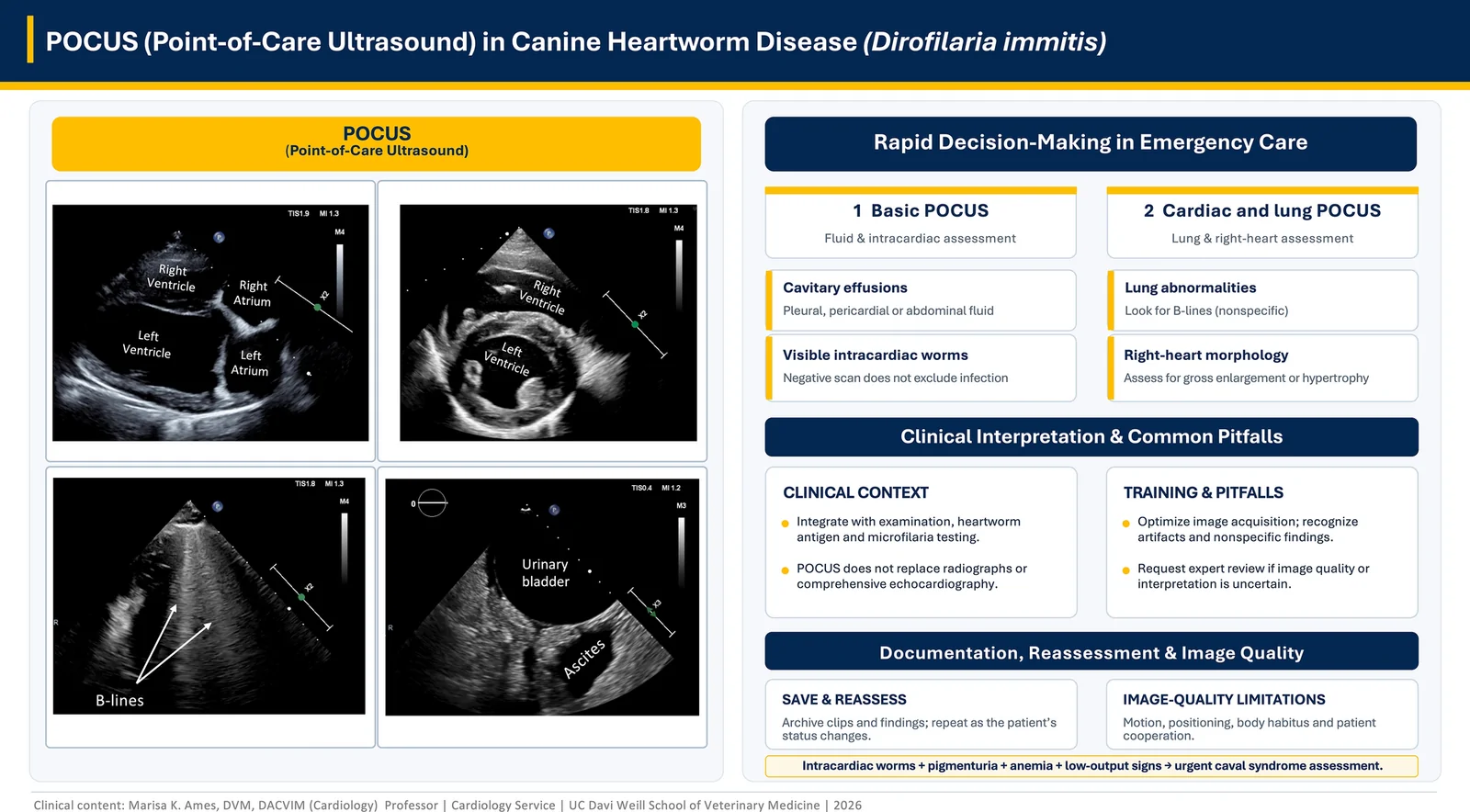

## Background

Dogs with chronic heartworm infection, large worm burdens, or high activity levels are more likely to exhibit severe clinical signs [1, 2]. Clinical signs of heartworm disease (HWD) typically reflect underlying pneumonitis, severe pulmonary hypertension (PH), right-sided congestive heart failure (R-CHF), or the presence of intracardiac heartworms. In dogs presenting with signs and examination findings suggestive of cardiopulmonary disease, point-of-care ultrasound (POCUS) enables rapid identification or exclusion of conditions requiring urgent intervention [3]. Because specialist availability, the dog’s stability, and cost often limit access to comprehensive echocardiography and full ultrasonographic studies, POCUS offers a practical, accessible alternative to support diagnostic and therapeutic decision-making. Although formal POCUS training is ideal, learning basic cardiac views can still help clinicians evaluate dogs presenting with cardiopulmonary signs. Established POCUS techniques include the thoracic and abdominal focused assessment with sonography for trauma (TFAST and AFAST) and veterinary brief lung ultrasound examination (Vet BLUE) [4–7]. Several veterinary ultrasound groups and universities offer formal training in POCUS for practicing veterinarians. The objective of this article is to briefly review the POCUS exam and demonstrate how POCUS is used when examining the dog with severe HWD. The clinical scenarios that will be reviewed include intracardiac heartworms, severe PH, R-CHF, pneumonitis, and miscellaneous scenarios.

## Point-of-care ultrasound

A recent survey of predominantly North American small animal practitioners found that 84% have an ultrasound unit in their practice [8]. Most of these practitioners (89%) had some formal training, yet the majority (52%) reported ≤ 25 h of training. The number of hours required to achieve proficiency in POCUS in veterinary medicine has not been determined, yet it is likely greater than 25 h. Guidelines for emergency ultrasound training for physicians recommend 25–50 quality-reviewed exams for each particular type of exam [9]. The aforementioned survey also identified a need and desire for more standardized ultrasound training for veterinary students. Training courses for veterinarians focus on techniques to assess the abdomen, pleural space, lung, and heart. Training requires time, on-going practice, and the costs associated with courses and equipment; resources which are not available to all practitioners. This review cannot overcome these obstacles or teach all the fundamentals of POCUS; rather, it will review its relevance to clinical scenarios that arise in dogs with HWD and hopefully inspire clinicians to seek out additional information and training.

## Terminology and technique tips

This review will use transducer (probe) locations as described by Lisciandro and colleagues [4–7, 10]. The reader is referred to these publications for useful diagrams and more in-depth descriptions of techniques. Microconvex transducers have a versatile footprint that can be used for both thoracic and abdominal POCUS examinations. Phased array transducers are excellent for intercostal cardiac windows. Linear transducers offer high resolution of superficial structures and are useful for imaging the lung. For examinations other than the cardiac exam, the transducer is generally oriented longitudinally (long axis aligned head to tail) and rocked and fanned at each location. The cardiac views rotate between long- and short-axis views of the heart. Ultrasound machines often have specific “presets” categorized by anatomic site and patient size. Many machines will have a cardiac preset where the screen marker, which corresponds to the transducer marker, on screen right. Images and transducer handling descriptions in this article use a cardiac preset with the marker on the right side of the screen.

The TFAST exam evaluates three sites on each side of the thorax (six total sites, as all views are bilateral): 1) the chest tube site at the caudal/mid-dorsal thorax to evaluate the interface of the visceral and parietal pleura (glide sign) and lung, 2) the pericardial site (PCS) at the cranial ventral thorax (over the heart) to diagnose pericardial and pleural effusions and evaluate the cardiac views, and 3) the diaphragmatic-hepatic (DH) site at the ventral thorax/cranial abdomen to diagnose pleural, pericardial, or peritoneal effusion and systemic venous hypertension (caudal vena cava and hepatic vein dilation) [11]. The cardiac apex beat is used to identify the approximate area of the PCS on the left side; the right PCS will be at that same level on the opposite side of the chest. For the DH view, the transducer is placed immediately caudal to the xiphoid and pointed cranially, so that both liver, cranial vena cava, and the heart are visualized. The Vet BLUE protocol evaluates the lung parenchyma at four sites on each side of the thorax: 1) caudodorsal, 2) perihilar, 3) middle, and 4) cranial [11]. These sites form a check mark shape when moving from cranial to caudodorsal regions. Three adjacent rib spaces are evaluated at each site. When evaluating lung with ultrasound, it is important to keep the long axis of the transducer oriented longitudinally (head to tail) and ensure consistent contact with the skin; however, when looking for small amounts of pleural effusion, rotating the transducer so that the long axis is aligned spine to sternum may be useful. With the transducer oriented, longitudinally, the anechoic rib shadows outline lung down the sector and the hemi circles at the top of the shadows (gator sign) indicate the expected level of the visceral/parietal lung interface.

**Fig. 1:**
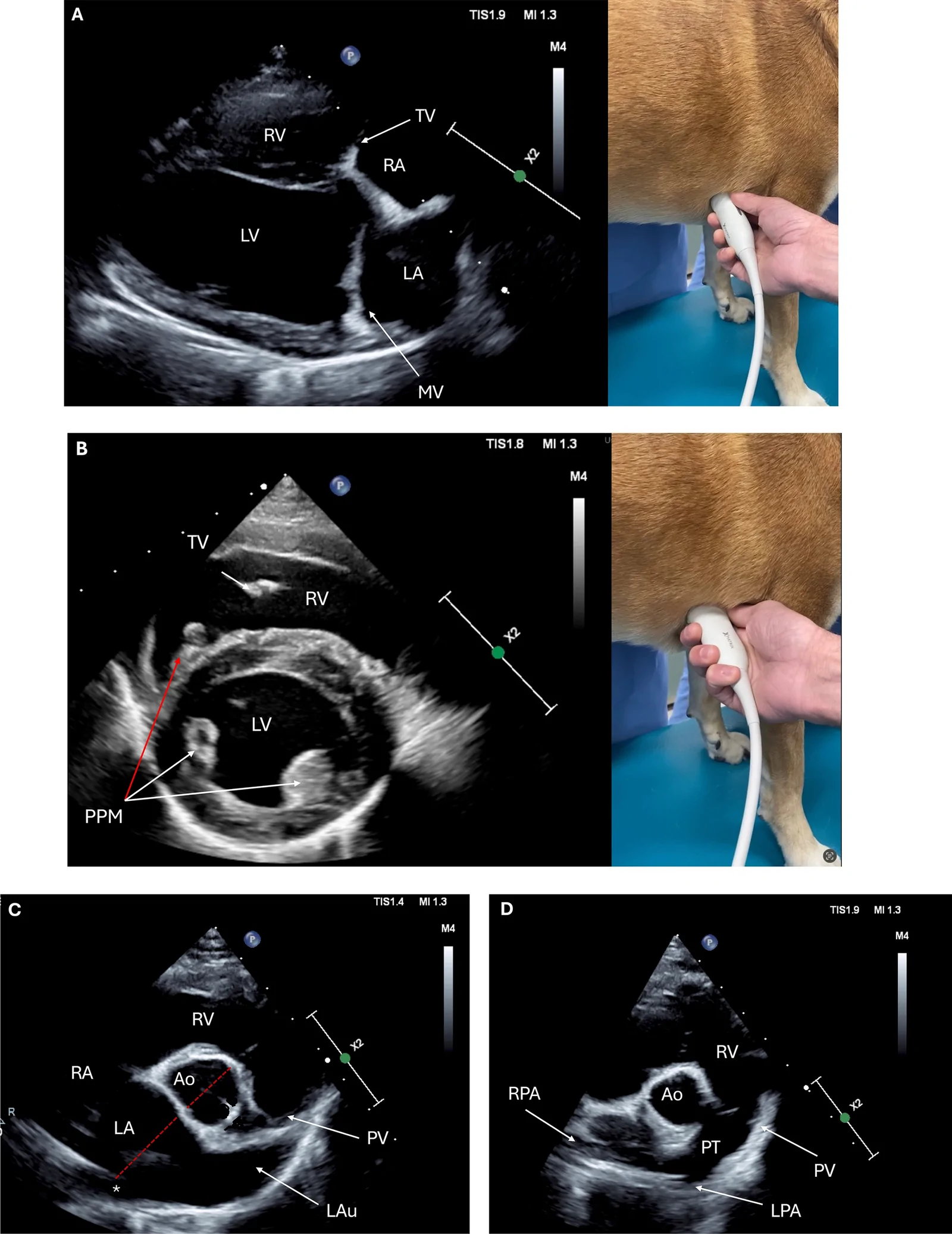
Four useful cardiac views (normal dog). **A** Right parasternal long axis—four-chamber view. The transducer is positioned with the marker (gray line under operator’s thumb) pointing toward spine (12 o’clock). Quality control includes ensuring all four chambers are in the view, and the left ventricle (LV) is not foreshortened (aim to make it as long as possible). **B** Right parasternal short axis (RPSSA) at the level of the LV papillary muscles (mushroom) view. From the four-chamber view, the transducer is rotated clockwise, so that the marker (under operator’s thumb) is at about 4 o’clock. Quality control includes ensuring the LV papillary muscles (PPM; white arrows) are symmetrical and that right-ventricular (RV) free wall in view (this sometimes require sliding the transducer dorsally). The PPM of the RV (red arrow) are also seen. **C** RPSSA left atrial-to-aortic view. From the mushroom view, fan the transducer (tail of the transducer goes down) dorsally to find the left atrial-to-aortic (LA:Ao) view. You may need to also slide the transducer dorsally and/or rotate clockwise to optimize the image. Quality control includes ensuring the aorta (Ao) is circular with leaflets visible. The red dashed line indicates where the aortic and LA diameters are measured; note the drop out (asterisk) in the LA wall, this is often due to a pulmonary vein confluence or the right branch pulmonary artery (RPA), a common cause of artifact, and a pitfall of imaging in this area; in this case, the LA wall’s approximate position is estimated based on the LA wall curvature. The triangular left auricle (LAu) should be visible. **D** RPSSA pulmonary trunk view. From the LA: Ao view fan the transducer cranially and dorsally to find the pulmonary valve (PV), pulmonary trunk (PT), and branch pulmonary artery views. Usually, the RPA is more easily visualized, whereas the left branch pulmonary artery (LPA) dives down and is hard to see. MV: mitral valve; RA: right atrium; TV: tricuspid valve

The most relevant cardiac views for dogs with HWD include the short-axis left ventricle (mushroom) view and the long-axis (four-chamber) view obtained from the right side. The four-chamber and mushroom views are fundamental to POCUS and should be accessible to clinicians with a basic training and experience. The short-axis left atrial-to-aortic ratio (LA:Ao) view, short-axis pulmonary artery view, and assessment of right-ventricular thickness and right atrial size on both long- and short-axis views require additional training and experience. These cardiac views are shown in Fig. 1.

The AFAST exam consists of 4 views: (1) DH view in the cranial abdomen, (2) splenorenal view in the left abdomen, (3) cysto-colic view in the caudal abdomen, and (4) spleno-intestinal umbilical view at the level of the umbilicus. Transducer placement for the splenorenal view is immediately caudal to the costal arch at its junction with the lumbar muscles [12]. Transducer placement for the cysto-colic view is the caudal abdomen lateral to the midline. Transducer placement for the spleno-intestinal umbilical view is at the level of the umbilicus. The DH view is helpful to visualize the caudal vena cava and hepatic vasculature. These views allow the clinician to screen for free fluid and distension of hepatic veins and the caudal vena cava, findings that support a diagnosis of R-CHF.

## Considerations and limitations

Point-of-care ultrasound is a useful tool for rapid decision-making in the emergency setting. However, POCUS findings must be interpreted in combination with results of the physical examination and appropriate diagnostic tests. Point-of-care ultrasound cannot characterize complex structural and functional abnormalities and is complementary to, but not a replacement for a comprehensive echocardiogram, abdominal ultrasound, chest radiographs, computed tomography, or magnetic resonance imaging. Training and experience are necessary to avoid the common pitfalls of inadequate image optimization and creation or misinterpretation of artifacts. Many of the ‘signs’ used in POCUS such as B-lines or the wedge-sign are non-specific and must be interpreted within the clinical context. New sonographers should ideally seek real-time or retrospective review of their images by an experienced sonographer. Point-of-care ultrasound captures a single moment in time and the exam should be repeated as the dog’s condition changes. Respiratory motion, the dog’s positioning (e.g., orthopnea), obesity, the dog’s compliance, can degrade image quality or obscure/mimic pathology. Finally, POCUS images should be archived and the clinician’s interpretations recorded in the medical record to maintain continuity of care.

## POCUS in the dog with heartworm disease: clinical scenarios

The scenarios below outline manifestation of severe HWD. Historical and examination findings, useful POCUS views, and treatment recommendations are presented. These clinical scenarios may prompt the clinician to perform a heartworm antigen test or represent urgent presentations in dogs with known heartworm infection. Dogs can have two or more of these manifestations of HWD occurring at once, as they are interrelated. For example, PH is usually present in dogs with R-CHF due to HWD [13]. Management recommendations will be included with the specific clinical scenarios but are not meant to be comprehensive.

### Intracardiac heartworms

Historical findings in dogs with intracardiac heartworms include those reflecting severe heartworm disease (exercise intolerance, lethargy, cough, dyspnea, inappetence), and those more specific to a moderate-to-large intracardiac worm burden (collapse, discolored urine, and pallor) [14]. Physical exam findings include a right-sided systolic murmur, increased lung sounds and/or crackles, and signs of caval syndrome (pallor, pigmenturia, prolonged capillary refill time, weak femoral pulses, cool periphery) [14]. The goal of POCUS in this clinical presentation is to look for intracardiac worms. Findings associated with severe PH or R-CHF may also be noted (see below for more information on these conditions).

Useful POCUS views include:

Cardiac exam: The right parasternal long-axis (four-chamber) and short-axis (mushroom) cardiac views allow for the assessment of intracardiac heartworms, which look like hyperechoic “equal signs” in the right atrium and/or ventricle, extending into the vena cavae. Whereas the four-chamber and mushroom views are considered basic views, with experience, the pulmonary trunk can be inspected for worms, and the right atrial size can be assessed.

**Fig. 2:**
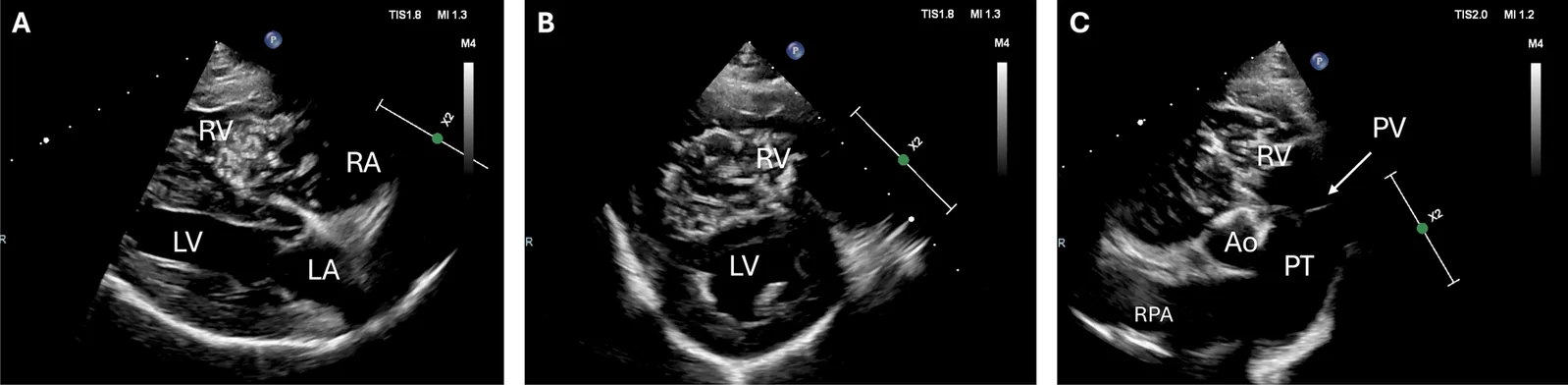
Cardiac point-of-care ultrasound from a dog that presented for lethargy, discolored urine, and abdominal distension. **A** Right parasternal long axis—four-chamber view. Intracardiac heartworms appear as “equal signs” within the moderately dilated right atrium (RA) and right ventricle (RV). Worms were prolapsing back and forth across the tricuspid valve. The worm cuticle creates these bright “equal signs” that are readily seen when the angle of intercept of the ultrasound beam is perpendicular to the worm. **B** Right parasternal short axis at the level of papillary muscles (mushroom) view. There is systolic flattening of the interventricular septum due to the RV pressure overload (PH). A mass of heartworms is seen in the RV. **C** Right parasternal short-axis pulmonary trunk view. The pulmonary trunk (PT) and right branch pulmonary artery (RPA) are markedly dilated, both exceeding the diameter of the aorta (Ao). A mass of heartworms is seen in the RV. This dog also had caudal vena caval and hepatic venous dilation and ascites (not shown). After stabilization, 81 heartworms were removed from the dog’s heart via trans-jugular venotomy. LV: left ventricle; PV: pulmonary valve

Intracardiac heartworms appear as hyperechoic “equal signs” (Fig. 2). Intracardiac heartworms and the finding of anemia, pigmenturia (hemoglobinuria), and signs of low cardiac output (prolonged capillary refill, cool periphery, weak arterial pulses, hypotension) confirm the diagnosis of caval syndrome. It is difficult to quantify the number of worms, yet when numerous, worms tend to form a tangle or mass that prolapses back and forth across the tricuspid valve with each contraction.

Whether to perform trans-jugular worm extraction in dogs *without* signs of caval syndrome is up to the clinician’s discretion and owner’s ability. Anecdotally, dogs with a large mass of worms in the right heart are at risk for developing anemia and hemoglobinuria and low-output heart failure of caval syndrome and worm removal is likely prudent. The anemia in dogs with high worm burden has been hypothesized to be due to mechanical trauma [15] and microangiopathic hemolysis due to fibrin trapping in disseminated intravascular coagulation, though data are lacking to prove the latter mechanism. In dogs with a single-exposure model of experimental heartworm infection, mean pulmonary artery pressure has been found to be significantly higher in dogs that develop caval syndrome as compared to dogs that do not develop caval syndrome [16]. Pulmonary hypertension is therefore thought to be one factor involved in the retrograde migration of heartworms in dogs with caval syndrome. Oxygen and sildenafil cause pulmonary vasodilation, reducing pulmonary vascular resistance, while pimobendan improves right-ventricular systolic function. In dogs with intracardiac heartworms, induction of anesthesia, and treatment with pimobendan and sildenafil with or without oxygen has been associated with migration of worms from the right side of the heart back into the pulmonary arteries [14, 17–19].

It is worth noting that linear artifacts such as the side lobe artifact which appears near reflective structures such as the pericardium or walls of the pulmonary arteries can be confused for heartworms. Artifacts are more likely to extend beyond the cardiac chamber or move independently from the cardiac motion.

### Severe pulmonary hypertension

The signalment and historical findings will vary based on the underlying cause of the PH. Diseases that lead to severe PH include heartworm disease, chronic pulmonary disease, cardiac shunts, and conditions of hypercoagulability that lead to thromboembolism [20]. Chronic, severe left-sided heart disease is a post-capillary cause of PH; however, in those cases, the left atrium will usually be enlarged on the LA:Ao view. Radiographic findings in dogs that are supportive of chronic PH include right-ventricular enlargement (“reversed D” on the ventrodorsal or dorsoventral view), dilation of the pulmonary trunk (bulge at one o’clock on the same view), and dilation (with or without truncation and tortuosity) of the branch pulmonary arteries.

Pulmonary hypertension may be clinically silent or it may cause many of the clinical signs and exam findings associated with severe HWD, including exercise intolerance, shortness of breath, cough, and syncope [21]. Chronic, severe PH is more likely to cause signs associated with R-CHF. Physical exam findings include cyanosis, tachypnea, dyspnea, increased lung sounds, pulmonary crackles, jugular venous distention, a ballotable fluid wave (peritoneal effusion), or muffled lung sounds (pleural effusion). A right-sided systolic murmur may be present. Possible causes of this murmur include tricuspid insufficiency due to right-ventricular remodeling, worms disrupting the tricuspid valve apparatus causing insufficiency, or concurrent degenerative tricuspid valve disease (small- to medium-sized, older dogs).

**Fig. 3:**
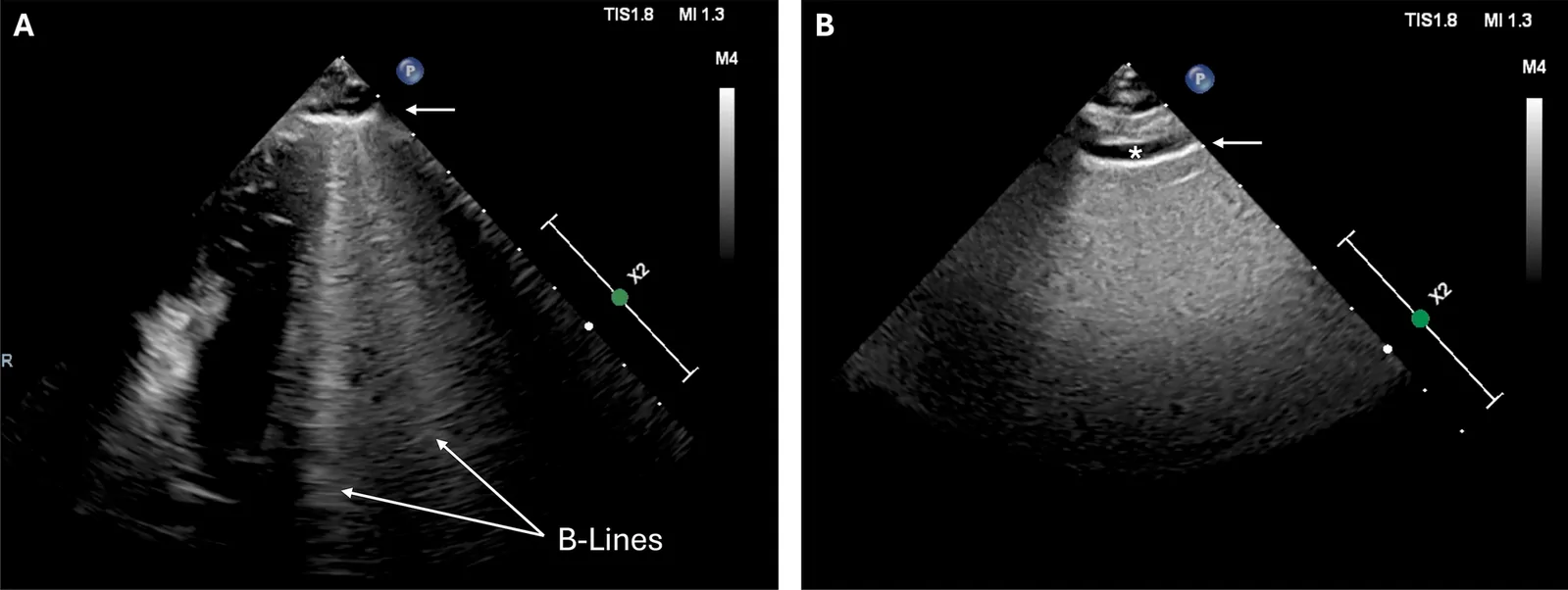
Lung ultrasound and B-lines. The visceral–parietal interface is indicated by an arrow at the top of the sector. **A** Dog with myxomatous mitral valve disease and cardiogenic pulmonary edema. Two vertical, hyperechoic lines (B-lines) extend from the visceral-parietal interface. These lines were noted to move synchronously with the lung. **B** Dog with HWD and presumed pneumonitis based on the finding of a severe, diffuse bronchial and interstitial pulmonary pattern and historical finding of a frequent cough. The lung is obscured by infinite B-lines. There is also a small volume of pleural effusion (asterisk)

**Fig. 4:**
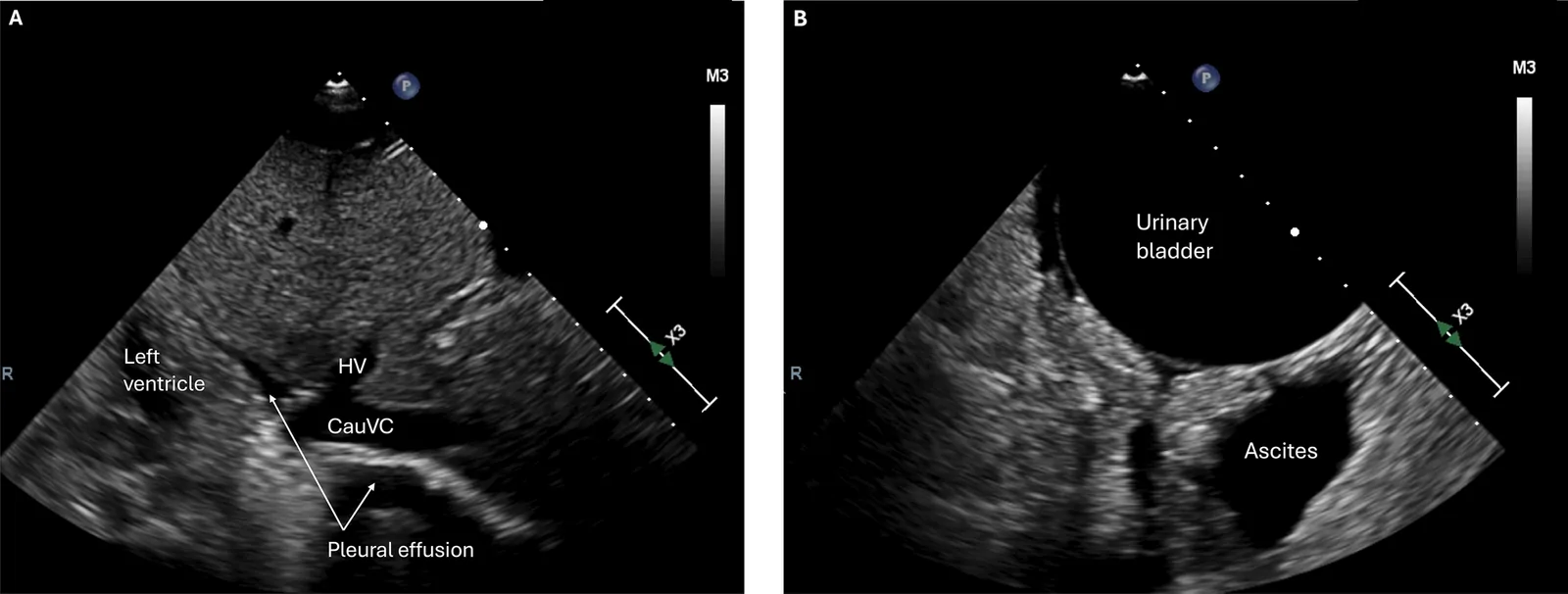
Diaphragmatic-hepatic view **A** and cysto-colic **B** views from the same dog in Fig. 3B. **A** The intraabdominal caudal vena cava (CauVC) is distended and noted to be non-distensible. The hepatic vein (HV) is also dilated. There is pleural effusion in the caudal thorax. **B** Anechoic free fluid in the abdomen (ascites) surrounds a distended urinary bladder. The presence of fluid on both sides of the urinary bladder makes its wall easily visible

Useful POCUS views include the following:

- Cardiac exam: Views include the right parasternal long-axis (four-chamber) and short-axis (mushroom) cardiac views. These views can be used to assess right-ventricular wall thickness and right-sided chamber dimension. With experience the pulmonary view, allows for evaluation of the pulmonary trunk and branch arteries (Fig. 1).
- Chest tube site and Vet BLUE views: These views evaluate the parenchyma and pleural space for abnormalities such as B-lines (non-specific finding supportive of interstitial abnormalities; Fig. 3), the wedge sign [22] (supportive of pulmonary thromboembolism), and pleural effusion.
- AFAST views: These views are used to evaluate for peritoneal effusion. The DH view allows for assessment of the caudal vena cava and hepatic vasculature (see R-CHF section; Fig. 4).

Left-sided congestive heart failure is a common differential diagnosis for animals presenting with respiratory distress. Left atrial enlargement is typically present in dogs with decompensated left-sided CHF. Because the LA:Ao usually exceeds 2:1 in dogs with left-sided CHF, a LA:Ao < 2:1 makes left-sided CHF less likely. [3]

In the absence of left-sided heart disease, right-sided chamber dimensions and diastolic wall thicknesses can be compared to the left side of the heart. The left and right atria are usually similar in size on the four-chamber view. The right-ventricular diastolic wall thickness should be thinner than that of left ventricle. The right-ventricular chamber dimension is normally about one-third that of the left-ventricular chamber dimension. Increased diastolic and systolic right-ventricular wall thickness (hypertrophy) is supportive of a chronic pressure overload, which is due to either PH or a right-ventricular outflow obstruction (e.g., congenital pulmonary stenosis). Pulmonary stenosis is the most common congenital heart disease causing right-ventricular hypertrophy in dogs. In affected dogs, a characteristic left-basilar systolic murmur is present from birth and persists throughout the life of the dog; if this murmur is absent, pulmonary stenosis is an unlikely differential diagnosis for right-ventricular hypertrophy. Pressure overload of the right ventricle also leads to flattening of the interventricular septum, usually during systole (Fig. 2B). Long-term studies evaluating the factors that influence the magnitude and durability of pulmonary pressure reduction and reverse cardiac remodeling after successful heartworm treatment are lacking. Dogs with R-CHF caused by HWD whose clinical sign improve or resolve with sildenafil and heart failure therapy can often be successfully weaned from these medications after successful heartworm adulticide therapy [17, 18].

More advanced sonographers should evaluate the pulmonary trunk and branch artery view. The diameter of the pulmonary trunk (just distal to the pulmonary valve) can be compared to the aortic diameter assessed at the LA:Ao view. The pulmonary trunk is often dilated in dogs with severe PH. Pulmonary trunk dilation is supported by a pulmonary trunk that is wider than the aorta. Pulmonary thrombus/thromboembolism is difficult to diagnose with ultrasound yet may appear as a hypoechoic wedge of tissue in the lung [22]. In rare cases, a thrombus is visualized in the right branch pulmonary artery. Sometimes these proximal thrombi include heartworm cuticle.

When chronic and severe, PH leads to R-CHF and resultant cavitary effusion (ascites and/or pleural effusion). In dogs with ascites, there is usually evidence of elevated systemic venous pressures (caudal caval and hepatic venous dilation, which is best seen on the DH view; Fig. 4). Pulmonary artery vasodilators, including sildenafil, tadalafil, or ambrisentan, are indicated in dogs with severe PH. In severe cases, oxygen therapy and sedation may be needed for initial stabilization, while sildenafil therapy is initiated.

Point-of-care ultrasound is not a definitive test for pulmonary hypertension. Even a comprehensive echocardiogram is only used to assess the likelihood that a dog has PH. POCUS is therefore best used to strengthen the recommendation for a comprehensive echocardiogram to more fully evaluate the probability of PH or evaluate an unstable dog that cannot immediately have a full echocardiogram performed.

### Right-sided congestive heart failure

Chronic, severe PH resulting from any cause, including HWD, causes right-ventricular dysfunction and ultimately R-CHF [20]. Obstruction from intracardiac worms leads to both low-output failure (reduced ventricular stroke volume) and R-CHF (cavitary effusions). Other causes of R-CHF include tricuspid valve insufficiency (usually at least a moderate-intensity right apical systolic murmur is present) and pulmonary valve stenosis (usually congenital and associated with at least a moderate-intensity left-basilar systolic murmur). Pericardial effusion and cardiac tamponade can cause a mechanical R-CHF that is reversible if the tamponade is relieved. Physical exam findings supportive of R-CHF include lethargy, shortness of breath, jugular venous distension ± pulsation, abdominal distension, ballotable fluid wave, and muffled lung sounds.

Useful POCUS views include the following:

- AFAST views: These views are used to evaluate for peritoneal effusion. The DH view allows for assessment of the caudal vena cava and hepatic vasculature (Fig. 4).
- Chest tube site and Vet BLUE views: These views evaluate the parenchyma and pleural space for abnormalities such as B-lines (supportive of interstitial abnormalities; Fig. 3), the wedge sign [22] (supportive of pulmonary thromboembolism), and pleural effusion.
- Cardiac exam: The right parasternal long (four-chamber) and short (mushroom) axis cardiac views are used to assess right-ventricular wall thickness and right-sided chamber dimension. With experience the pulmonary view, allows for evaluation of the pulmonary trunk and branch arteries (Fig. 1).

Fluid such as blood or effusion is anechoic (black) on ultrasonographic images. Fluid is normally only seen within the cardiac chambers, vasculature, gallbladder, gastrointestinal tract, and urinary bladder. Fluid outside of these spaces is considered abnormal (Fig. 4). Cavitary effusions can be seen on any of the POCUS views, though only with increasing severity in the non-gravity dependent regions (dorsal regions when the dog is standing or sternal). Fluid often forms triangles or other angular shapes when mild to moderate in severity. Severe effusion is readily apparent. An AFAST fluid scoring system has been described [4]. Cardiogenic effusions arise from right-sided dysfunction and increased right-ventricular filling pressures. Support for a cardiogenic cause of cavitary effusions includes jugular vein distension, right-sided heart enlargement, dilated caudal vena cava, dilated hepatic veins, and fluid analysis results indicating a modified transudate. The diameter of the intraabdominal caudal vena cava generally increases on expiration and decreases on inspiration; a distended caudal vena cava with reduced collapsibility (< ~ 30%) is supportive of increased systemic venous pressure [23]. Pericardial effusion may occur secondary to R-CHF, though is usually small volume and without cardiac tamponade. Pericardial effusion conforms to the contour of the pericardial space, often appearing as an anechoic ring around the myocardium bound externally by a hyperechoic (white) pericardium. Point-of-care ultrasound criteria for cardiac tamponade include diastolic collapse of the right atrium and sometimes right ventricle concurrently with caudal vena cava distension and reduced collapsibility.

Semi-quantitative assessment of fluid volumes and the dog’s clinical signs are considered when deciding whether to perform centesis. Mechanical removal of moderate or greater volumes of effusion is ideal. Diuretic therapy and ancillary cardiac medications help prevent or slow the recurrence of effusion yet usually cannot resolve large fluid accumulations.

Several limitations should be considered. Once cavitary effusion is identified, further evaluation is necessary to determine the cause. Additional diagnostic tests include a comprehensive physical examination, fluid analysis with cytology, and advanced diagnostic imaging (radiographs, comprehensive echocardiogram, abdominal ultrasound). Point-of-care ultrasound does not allow for quantification or characterization of fluid. Ultrasound cannot quantify fluid yet can help the clinician decide about the need and feasibility of diagnostic or therapeutic fluid removal.

### Pneumonitis

Lung ultrasound can support the diagnosis of pneumonitis, which is a common cause of cough in dogs with HWD.

Useful POCUS views include the following:

Chest tube site and Vet BLUE views: These views evaluate the parenchyma for abnormalities such as B-lines (Fig. 3).

Pneumonitis secondary to HWD may lead to B-lines, which are hyperechoic lines originating at the visceral/parietal interface that radiate vertically down the sector and move synchronously with the sliding lung. One common differential for B-lines in the dog with respiratory distress is cardiogenic pulmonary edema. In this case, the left atrium is usually also enlarged (e.g., LA:Ao > 2.0). The glide sign (lung sliding) is the movement of the visceral lung surface relative to the more fixed parietal surface. In the absence of anything within the pleural space, these two surfaces abut one another and ‘slide’ back and forth as the animal breathes, creating a shimmering line [24].

Lung ultrasound relies largely on recognizing specific artifacts, which requires training and experience. B-lines are non-specific, resulting from any cause of either increased lung water or cellular infiltration, and findings from the physical exam and appropriately selected additional diagnostic tests are necessary to determine the cause of B-lines. Lung pathology must also be located at, or within millimeters, of the visceral surface of the lung to be best visualized with ultrasound. Pathology occurring away from the surface of the visceral pleural cannot be visualized with ultrasound. For these reasons, lung ultrasound does not replace thoracic radiography.

### Miscellaneous scenarios

Lung ultrasound can also support a diagnosis of pneumothorax, which is occasionally noted in dogs with HWD. Pneumothorax is supported by the loss of the glide sign or the presence of B-lines that are seen originating at areas distinct from the visceral/parietal interface. Pulmonary granulomas are rarely reported in dogs with HWD and may lead to a nodule sign (hypoechoic lesion with a hyperechoic shadow).

## Conclusions

Point-of-care ultrasound is more accessible than echocardiography and can help the clinician assess the dog with HWD. Severe clinical signs usually arise from PH, R-CHF, or the presence of intracardiac heartworms. Radiographs and POCUS are useful tools that complement one another and help refine the differential diagnosis list when evaluating the dog presenting with cardiopulmonary signs. Finally, POCUS findings should be viewed as supportive and must be interpreted in combination with results of the physical examination and appropriate diagnostic tests.

## Data Availability

Data supporting the main conclusions of this study are included in the manuscript.

## Abbreviations

AFAST: Abdominal focused assessment with sonography for trauma
DH: Diaphragmatic-hepatic site
HWD: Heartworm disease
LA:Ao: Left atrium to aortic ratio
PCS: Pericardial site
POCUS: Point-of-care ultrasound
PH: Pulmonary hypertension
R-CHF: Right-sided congestive heart failure
TFAST: Thoracic focused assessment with sonography for trauma
Vet BLUE: Veterinary brief lung ultrasound examination
